# Behavioural Engagement of Holstein Friesian Dairy Cattle with Different Mounting Techniques for Salt Licks as Environmental Enrichment

**DOI:** 10.3390/ani15050701

**Published:** 2025-02-27

**Authors:** Danielle Lauren McLaughlin, Nicola Blackie

**Affiliations:** Pathobiology and Population Sciences, Animal Welfare Science and Ethics Group, Royal Veterinary College, Hatfield AL9 7TA, UK; dmclaughlin20@rvc.ac.uk

**Keywords:** dairy cattle, environmental enrichment, salt lick, positive welfare, behaviour

## Abstract

With increasing numbers of dairy farms adopting zero-grazing systems, there is a growing need for indoor environmental enrichment methods. Enrichment is necessary to meet industry requirements and fulfil behavioural motivations, such as oral manipulation. This study evaluated the magnitude with which Holstein Freisen cows would interact with salt lick enrichment blocks based on the mounting design. Holstein Freisen dairy cows (n = 55) were recruited from a UK dairy farm and observed over a 4-week period (n = 20 days). Three different mounting designs were utilized, low non-moveable (LNM), low moveable (LM), and high moveable (HM), and the LNM setup was repeated on week 4. These mounting designs were each observed over a five-day period and then removed for two days in-between. Data were collected by in-person observation and included cow IDs, instances of interaction, and kilograms of salt lick used per setup. The data were analysed through IBM SPSS Statistics via a One-Way Repeated Measures ANOVA and Microsoft Excel to determine significant findings and habituation. The number of new interactions significantly decreased in the HM setup compared to the LM and LNM. The supporting data of kilograms of salt lick used and total percentage of the herd utilizing the blocks, also favoured the LM setups over LNM. The LNM setup was repeated on the final week to assess the level with which cows had habituated to the environmental enrichment. Despite a significant difference between week 1 and week 4, the trends of cow interactions showed individual variability in habituation and overall negligible herd-level habituation. These findings suggest that the use of mineral licks within a dairy herd serves as effective environmental enrichment, even over extended time periods, and when implemented they are best used at low heights with the ability to have free movement. When implemented on a farm, the LM mounting design should increase the herd-level uptake of enrichment leading to a reduction in stereotypies and fulfilment of oral motivation, which is beneficial for overall cow health and welfare.

## Simple Summary

During this study, a UK dairy herd was observed in order to gain further understanding into how design strategies for environmental enrichment alter overall herd engagement. This was assessed by altering mounting setups for salt lick blocks and measuring how the cattle then interacted with them. The setup treatments were low freely hanging blocks, low stationary blocks, and high moveable blocks. This study examined whether these mounting techniques influenced individual cattle and herd interactions. It was found that the total number of new interactions were significantly reduced in the high moveable blocks, but greatly increased with low moveable blocks. It was also found that the overall consumption of salt lick, in kilograms, was significantly increased in the low moveable setup. These findings support the conclusion that salt licks hung to freely move at low heights will maximize the uptake of environmental enrichment. This will then give farmers more efficient ways to increase the overall welfare of their animals and reduce the instances of stereotypies seen within the production animal industry.

## 1. Introduction

### 1.1. Industry Standards

In the UK, it is currently estimated that 20% of dairy herds have adopted zero-grazing systems [1] where cattle are housed indoors throughout their lifetime [2]. Keeping animals indoors can provide certain welfare benefits, including protection from predators and the weather [3], reduced exposure to toxic plants and parasites, and a further ability to provide a nutritionally complete diet for high-yielding cattle [4,5].

Indoor housing forces animals to make substantial changes to their daily time budgets [6]. To address this, the farming industry in the UK, under guidance by the Department for Environment Food and Rural Affairs (DEFRA), has laid out a Code of Recommendations for the Welfare of Livestock. This document outlines both legal requirements, such as the Five Freedoms, and best management practices in farming all life stages of bovine species (2003). Alongside government legislation, many assurance schemes, such as Arla, Red Tractor, and RSPCA-assured, have further welfare requirements for farmers to adhere to in order to be considered under the specific label (Red Tractor) or cooperative (Arla). The benefits of joining such schemes include increases in business growth opportunities, enhancing the farm’s reputation, and cutting down on individual farmer’s paperwork and costs [7,8,9,10].

### 1.2. Defining Enrichment

When discussing welfare and enrichment, a general definition of the term and the associated expectation are needed for changes in animal management to be equal and comprehensive from farm to farm. Welfare is considered beyond the absence of harm but also the promotion of positive emotive conditions [11]. This can be achieved through excellent management and further enhanced by environmental enrichment. The fundamental goal of enrichment is the fulfilment of species-specific behaviours that general management may not take into account [12]. Enrichment can then be further broken down into specific categories such as social (e.g., group housing calves [13,14,15] and dam rearing [16,17,18]), physical (e.g., wall partitioning [19] and fresh bedding [20]), environmental, occupational (e.g., exercise yard access [21,22]), sensory (e.g., the addition of mirrors [23] and automatic brushes [24]), and nutritional (e.g., nutritive rubber teats for calves [25,26]). For this study, the categories of importance are the environmental, defined as modifications made to management styles or the physical circumstances of the animal that undoubtedly improve biological functioning [6] or other welfare measures going beyond minimum management standards [27] and its sub-group, physical enrichment. Physical enrichment is defined as altering the size or complexity of the animal’s enclosure or adding accessories to the enclosure such as objects, substrate, or permanent structures [28].

### 1.3. Oral Stereotypies

The food searching and eating times of cattle normally extend from 6 to 12 h on pasture, but are reduced to 4 h per day indoors [29]. This leaves a significant gap in time, which must be occupied. The lack of environmental stimuli may contribute to frustration and stereotypies related to their inability to satisfy motivations, such as oral manipulation [13].

Stereotypies can be displayed as leg stamping [30], non-nutritive oral fixation, like tongue rolling [31], and the visualization of eye whites [32]. A study conducted in 2000 by Lindstrom and Redbo [33], characterized the effect of feeding duration and total rumen fill on behaviour patterns in dairy cows. They found that “oral manipulation of feed is a behavioral need in cattle irrespective of rumen load [33]”. It can then be concluded that the practice of low feeding duration does not allow for the expression of oral manipulation over long periods and could impair the welfare of cattle. Enrichment is then employed to satisfy these specific behaviours and lower the instance of stereotypies while increasing overall welfare. In this study, the enrichment used was the implementation of mineral blocks throughout the yard.

### 1.4. Mineral Licks

It is important to note that when considering the mineral lick as a form of enrichment, the mechanism that underpins its success must be defined [13], but that enrichment can be multifactorial as well, having both short- and long-term effects. A mineral lick added to an indoor housing system encompasses physical, environmental, sensory, and nutritional enrichment. A mineral lick would be an added accessory to the cow’s enclosure, provide a modality of oral stimulation including taste and touch, be a short-term visual addition which peaks investigation, and add a new locus of nutrient delivery deemed to be novel in the short-term. This categorical description was extrapolated from the enrichment review by van de Weerd and Day [34] on pigs.

Alongside the potential benefits to cattle welfare, mineral licks are a source of macro- and micro-nutrients required for a range of physiological functions. The most essential being salt, which has a role in cow growth, production efficiency, and fertility. Insufficient dietary sodium has been linked to reduced feed intake, drops in milk yield, and lower conception rates [35]. A study by Grewal [36] looked at the effect of free-choice mineral blocks on milk production and found that the use of these supplements increased the milk yield of individual cattle by 2 kg per day. Compared to the control group, these cattle also had increased milk fat and protein content. Enrichment studies have shown preferences regarding the placement and movement of items. DeVries [37] demonstrated that cattle prefer automatic rotating brushes over fixed brushes as thise allowed for the grooming of areas that were previously unreachable and provided cattle with more opportunity to display grooming behaviours. Another study, by Bulens et al. in 2014 [38], encouraged play behaviour in calves by hanging brushes or balls at a height of 1.3 m and found it to be highly beneficial in socially isolated animals. It is hypothesized that when given the option, cattle will show a significant increase in engagement with the elevated free-hanging mineral block compared with other block heights and fixture adaptations.

### 1.5. Study Aims

The goal of this study is to determine if the way a mineral block is installed leads to preferential behaviour and engagement. Our investigation aimed to evaluate differences in item engagement when placed at ground level versus elevated and when the block is stationary versus free-hanging.

## 2. Materials and Methods

### 2.1. Animals and Ethics Review

This project was undertaken on a housed Holstein Friesian dairy farm in the East of England. All procedures carried out were approved by the Clinical Research Ethical Review Board at the Royal Veterinary College (URN CR-2024-001-2) and consent was given by farm staff members in writing. Holstein Friesian cows (n = 55) were observed on a farm with minimal disruption to normal husbandry and daily milking routines.

The study was carried out on a loose-housed dairy farm in the East of England consisting of two yards for lactating cattle: one for high yielders and one for low yielders. Bedding was sawdust which was replenished daily and there was concrete next to the feed barrier. Cows were fed a TMR (total mixed ration) tailored to each group’s lactation stage daily, available upon return from morning milking. The ration was robotically pushed up every hour. Additional concentrate feed was given in the milking parlour. Milking occurred twice a day at approximately 5 a.m. and 3 p.m.

### 2.2. Building and Block Setup

**Figure 1:**
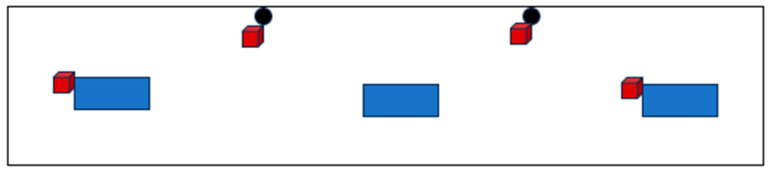
High-Yielding Barn with Block Setup. The above figure is a drawing of the high-yielding pen from an aerial view. Blue blocks represent water troughs. Black circles represent cement posts positioned on the back wall of the barn. The red blocks indicate the location of the salt licks. The blocks were labelled 1–4 corresponding with the blocks, respectively, from left to right. This setup was relevant to weeks 1, 2, and 4.

**Figure 2:**
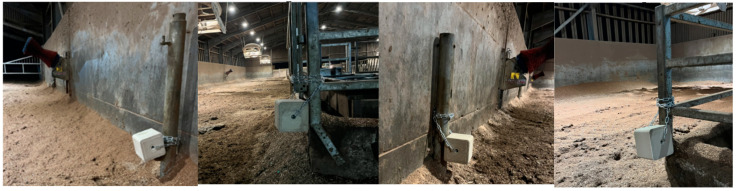
This figure shows the exact setup of the salt licks from day 0–4 and day 20–23 of the study. The blocks were labelled 1–4 as seen from left to right, respectively.

Maxcare minerals (Trouw Nutrition GB, Ashborne, UK) for cattle in 10 kg blocks (n = 4) were installed in the high-yielding cattle yard as seen in Figure 1. The blocks were secured with individual 2 m lengths of short link chain 5 mm in diameter and carabiner clips. For week 1, the mineral blocks were installed 30 cm from the ground in a low non-moveable (LNM) position, as in Figure 2. These mineral licks were removed for a two-day period at the end of week 1 (on day 4) and weighed. This rest period served to mimic the novelty factor in cow interactions with the lick upon each new block placement in subsequent weeks. 

**Figure 3:**
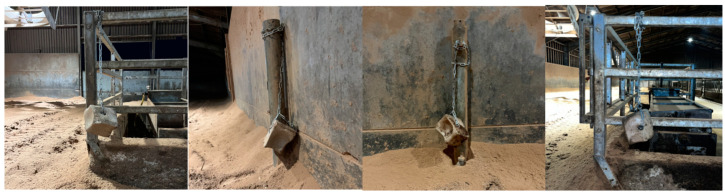
This figure shows the exact setup of salt licks from day 7–11 of the study. The blocks were labelled 1–4 as seen from left to right, respectively.

On week 2, day 7, new unused Maxcare blocks were re-installed, before morning milking, at the same location (Figure 1) and a height from the ground of 30 cm. However, the blocks were free hanging on 1 m of chain to allow movement, Figure 3. This treatment was defined as low moveable (LM). The blocks were removed for a two-day period at the end of week 2 (on day 11) and weighed.

**Figure 4:**
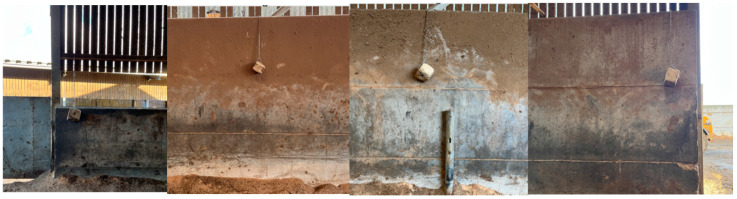
This figure shows the exact setup of salt licks from day 14–17 of the study. The blocks were labelled 1–4 as seen from left to right, respectively.

On week 3, day 14, new unused Maxxcare blocks were installed in the high-yielding barn, Figure 4. Blocks number 1 and 4 had to be relocated to the left outer wall and right outer wall, respectively, due to hanging access within the barn. The blocks were suspended on 1 m of chain to allow movement and were at a height of 150 cm from the ground. This treatment was defined as high moveable (HM). The blocks were removed for a two-day period at the end of week 3 (on day 17) and weighed.

**Figure 5:**
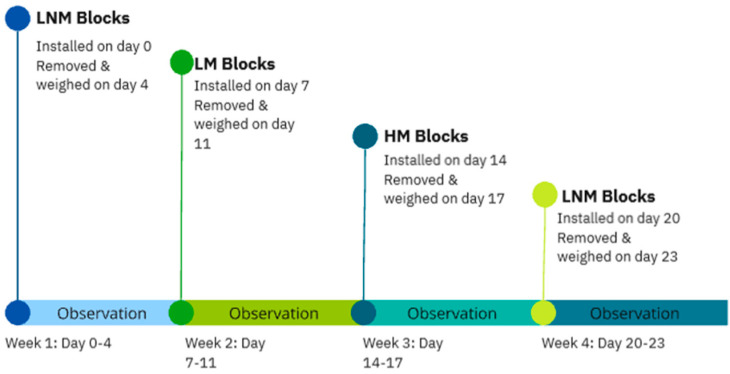
Timeline of the Study.

Table 1 summarises the salt lick mounting setups used in the study and the timing. Figure 5 shows the timeline of the observations 

### 2.3. Behavioural Observations

The behavioural observation of the cattle was carried out by a single observer who remained the same throughout the study to minimize subjective bias. No intra-observer reliability measures were undertaken, though all observations were based on the definitions in Table 2. Observations were carried out in a scanning method, in which the observer spent 1 min at each block, in the order from 1 to 4, for a period of an hour. This was repeated three times a day at 8 a.m., 11:30 a.m., and 2:30 p.m. This method was then repeated each day for 4 weeks (Figure 5), which amounted to 60 h of observation in real time on the farm and 900 observation points per mineral block. During the final week, week 4 of the study, on-farm construction led to the loss of a single day of data. This was accounted for later upon statistical analysis.

During observation, the number of interactions and the individual cow IDs were recorded manually. As data were collected, new interactions were identified and used later in data analysis. Further observations that were noted, were instances of agonistic behaviour around the blocks and individual cow interactions.

### 2.4. Statistical Analysis

All statistical tests were completed on IBM SPSS Statistics Data Editor (v30) and Excel. Raw data were inputted into Excel and daily means of new interactions, total individual cow interactions, and kilograms of salt licks used per week were calculated. Bar charts displaying averages were compiled in Excel.

A One-Way Repeated Measures ANOVA was used for instances of interactions data due to the parameters of this project, using the same individuals to test a continuous dependent variable. This tested for a significant statistical difference between the mean number of interactions per salt lick each week set up within the same herd. For each lick design, the mean number of interactions each day was used, as in n = 4, per week. The study originally followed cows 5 out of 7 days per week; however, due to interference, a day of data on week 4 was lost and, therefore, the same day from previous weeks was cut from the analysis.

To undertake an ANOVA, three assumptions, regarding the data set gathered, needed to be fulfilled. Those assumptions were: the data are normally distributed, there are no outliers, and the data are spherical. Boxplots were created through the “Explore” function in SPSS to identify any outliers amongst the data set. The Shapiro–Wilk test for normality was used to determine the distribution of the data. Sphericity was tested using the Mauchly’s Test of Sphericity and occurred with the ANOVA. Supporting data (i.e., kilograms of salt ingested and the percentage of herd interaction with licks) were plotted in Excel as standard bar graphs.

## 3. Results

### 3.1. Assumptions

**Figure 6:**
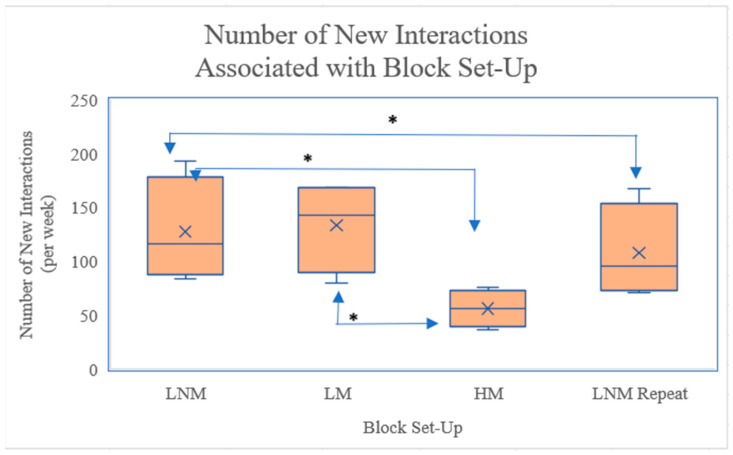
Box and Whisker Plot. Figure 6 illustrates the pairwise comparison between the mean number of cow interactions and the block setups each week from the ANOVA analysis. * = *p* < 0.05.

Before undertaking a One-Way Repeated ANOVA, the assumptions that the data are normally distributed, there are no outliers, and the data are spherical, must be confirmed. No outliers were found on inspection of the boxplot, as in Figure 6. The assumption that the mean number of interactions were normally distributed could be confirmed if *p*-values were >0.05. The Shapiro–Wilk test of Normality indicated that there is not enough evidence to deem the data non-normal, as all *p*-values are above 0.05. Sphericity, however, is accepted with a *p*-value < 0.05. The Mauchly’s Test of Sphericity found the assumed sphericity of these data is 0.012.

### 3.2. ANOVA

Figure 6 demonstrates that LNM had a significantly higher number of interactions compared to HM and LNM (repeat), *p* < 0.05. LNM had a mean difference of 71.25 new interactions when compared to the HM setup. The LM setup was also significantly increased in the number of new interactions when compared to HM, *p* < 0.05.

**Figure 7:**
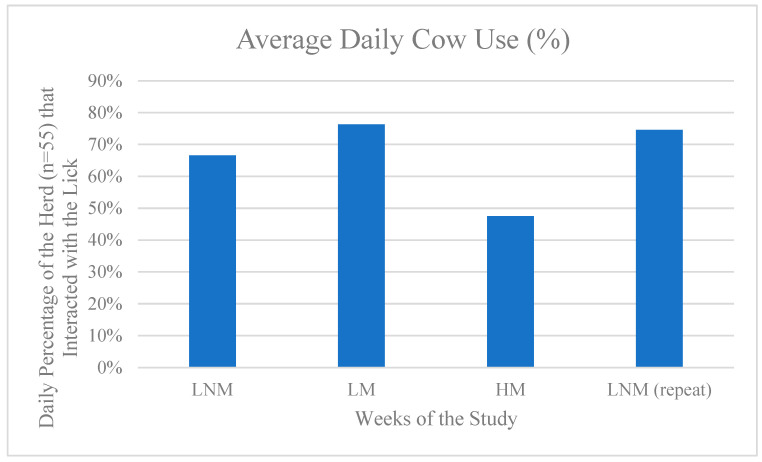
The average daily cow use of salt licks. Figure 7 shows the average percent of the total herd that interacted with the salt blocks daily depending on the setup.

Figure 7 visualizes the true number of cows within the high-yielding herd that interacted with the licks rather than instances of interactions. The average number of cows within the herd that interacted with the lick daily was highest with the LM blocks. The LNM and LNM (repeat) were only 9.8% and 1.8% lower, respectively, to the LM blocks. However, the HM setup was used by 28.8% fewer cattle than the LM setup.

**Figure 8:**
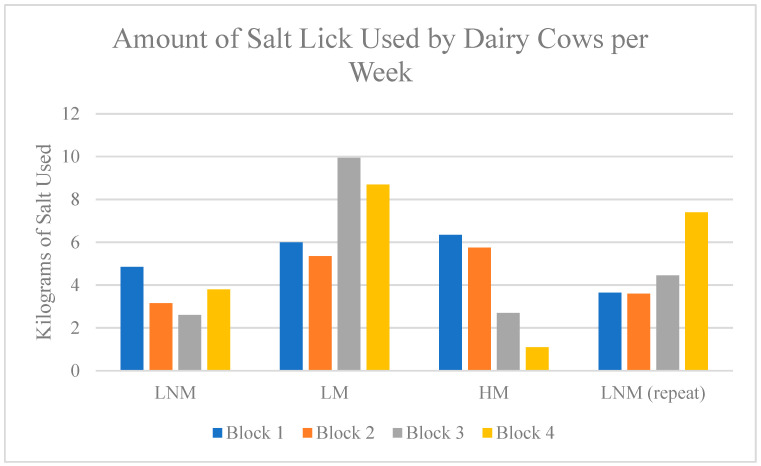
Amount (in kg) of salt lick used weekly. Figure 8 shows how much salt was ingested each week of the study and is broken up further into the individual salt blocks spread throughout the yard (blocks = 4). The block numbers correlate to the numbers assigned in the methods section.

In Figure 8, the distribution of salt used paired with the block numbers and the respective block setups are displayed. This shows the sharp increase in uptake with LM blocks, specifically blocks 3 and 4. Also, cattle used approximately 30 kg of salt lick during the LM setup. In comparison to the other weeks, this was the highest amount by 10.9 kg. The week 1 LNM, had the lowest uptake of salt at 14.4 kg, a difference of 15.6 kg.

**Figure 9:**
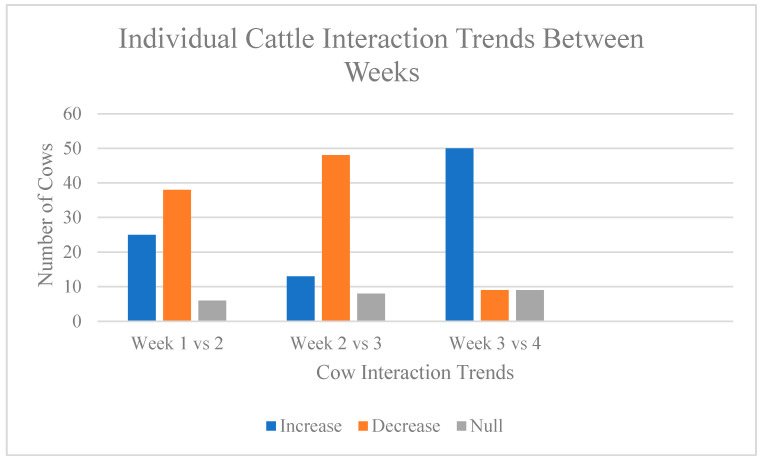
Individual Cattle Interaction Trends Between Weeks. Figure 9 shows how many cattle either increased, decreased, or stayed the same in the number of individual interactions compared week to week.

Individual cow ID tags were also used to determine on how many days of the study (n = 20 days) a cow utilized at least one salt block. The number of days ranged from 1 to 17, out of 20 available days, with a mean of 9 days per cow, a median of 10 days per cow, and a standard deviation of 4.4. The top five cows, which used the blocks at an almost daily consistency can be seen in Table 3. The same top five cows were found to have the highest number of total interactions, which was measured as the number of times a cow was seen at the block, regardless of if they had been seen on the previous observation point, Table 4. The highest number of total interactions was 118, compared to the least amount of 1. The mean number of total interactions was 36 with a median of 30, which shows significant uptake in the top five cows. Interactions with the blocks were also assessed based on trends between weeks, whether interactions increased, decreased, or remained the same (null). It can be seen in Figure 9 that between week 2 (LM) and week 3 (HM) 48 cows decreased the amount in which they interacted with the blocks. However, from week 3 (HM) to week 4 (LNM repeat) there was an inversely proportional number of cows which increased the uptake of the blocks, resulting in a 50-cow increase.

Lastly, individual cow IDs were noted upon any instance of aggression towards another cow, pertaining to usage of the blocks. These aggressive interactions were compiled in terms of the week in which they occurred, the block setup, and the descriptive nature in Table 5. Aggression was seen at its peak on week 1 with the LNM setup, with 13 instances of aggressive behaviour. This then drastically declined for the remainder of the study and did not increase again upon the LNM (repeat) setup, on week 4.

Table 4 displays the top five individual cows that interacted with the block on the most days over the study (n = 20 days).

Table 5 displays the top five individual cows that had the highest number of individual interactions over the entirety of the study.

Table 5 displays the amount and form of agonistic interactions between cattle using the salt blocks.

## 4. Discussion

### 4.1. Significant Findings

This study demonstrated that cattle interacted with salt licks significantly more when they were positioned at a low level compared to high. There was also an increase when the lick was freely moveable. A study on environmental enrichment in pigs showed cortisol levels in pigs were lower when interacting with a fixed floor object compared to items which were free hanging at shoulder height [39]. Although the heights used were different to the present study, the enrichment object in relation to the height of the animal is the same. There was a significant decline in instances of use and overall lick consumption when positioned 150 cm from the ground. Along with fewer overall cows within the herd utilizing the enrichment when it was placed at 150 cm. This could be due to the abnormal head and neck extension required to reach the items compared to low height installments, which mimic normal grazing positions. This could also be explained by the laterality of cattle vision [40], which allows for efficient scanning of the surrounding area, but may mean their tendency to look upwards is lessened. Therefore, not only was accessing the high-hanging blocks more difficult, but also for smaller cattle simply visualizing the blocks may have been challenging.

There were no significant differences found for instances of use between the low moveable and low non-moveable setups. However, there was a marginal increase in the mean instance of new interactions alongside supporting data which showed that both the amount of lick consumed and percentage of the herd using the enrichment were increased when the licks were free hanging. Frequently, free hanging balls are used as enrichment for calves to fulfil butting and play behaviour [38]. So, in this way, the increased overall use could be attributed to play behaviour or the ability to engage in further social behaviours alongside fulfilling masticatory needs. Increased use of a free-hanging rope was also seen in a study of calves in which the animals spent longer time periods orally manipulating a manila rope versus brushes, but also varied greatly in usage between animals (i.e., some calves used the rope constantly whereas others barely interacted with it). This study hypothesized that usage of the rope differed due to a calf’s motivational needs, social facilitation, and resource guarding [41], which could be applied to the cows in this study who showed great variance in the uptake of enrichment.

Most behavioural problems relating to aggression in cattle seem to be generated by boredom [42]. This study had the highest number of agonistic behaviours on week 1, when the first enrichment items were introduced. However, from week 1 to 2, these behaviours decreased to approximately 1 per day and did not increase when the blocks were changed. As the study was focused on enrichment, the potential positive effects on stress and time budgeting for oral manipulation could have decreased boredom and therefore aggression. Also, in 2014, it was observed that less aggressive and submissive behaviours were observed with increased inter-cow distances [43]. The yard design was arranged to maximize distance between blocks to allow cows ample space and availability, which may have helped to decrease aggression and resource guarding once the novelty of the objects decreased.

### 4.2. Habituation

Habituation is defined as, “the diminishing of an innate response to a frequently repeated stimulus” [44]. In this study, habituation was assessed in two ways: by analysing the weekly trends of enrichment interactions and by repeating week 1 of the study in week 4 to directly compare figures. The outcomes of this were vital as the resources put into enrichment attempts are economically unviable and of limited benefit if the cattle rapidly habituate to the salt licks [45].

The data collected are unclear overall on the level at which habituation occurred. When simply comparing the number of interactions between week 1 and 4, there is a significant decrease (n = 19, *p*-value < 0.005) in week 4. This suggests that habituation to the licks did occur and, therefore, the viability of the enrichment must be questioned. However, supporting data, such as the kilograms of salt consumed and the percentage of the herd who interacted with the block, were both higher in week 4. In week 4, approximately 5 kg more of the salt licks were consumed and 8% more of the herd took part in using the enrichment. Also, there was an increase in cattle usage of 50 cows from week 3 to 4. The way in which interactions were counted may have a part in this. If cattle were staying at the licks for longer, but returning less throughout the day, this would account for the increased ingestion. This would also lead to less competition around the lick and allow more opportunities for individual access. In 2006, Van de Weerd et al. [34] found that animals will sustain interest in preferred resources when that enrichment is inherently reinforcing, such as a food item. Therefore, though this may resemble habituation, it could be simply an increase in the length of time spent with the item, which was not recorded in this study.

Lastly, cows’ habituation to enrichment items has previously not appeared as a consistent group-level response [46]. Therefore, for future studies it may be pertinent to look at habituation on an individual level, especially as there was large variability between individual usage in this study. Data such as time spent with the enrichment item alongside the sheer number of interactions should be included. In the case of this study, given there was still an increase in use from week 3 to week 4 and supporting data between week 1 and 4 show active usage of the enrichment items, it was concluded that significant habituation to the items was not observed. A further study could assess whether the removal of the blocks for periods of time then reinstating them would decrease habituation and encourage more engagement in the enrichment.

### 4.3. Application

In housed dairy cattle, potential welfare concerns surrounding captivity, such as stress, boredom, and the lack of an ability to express highly motivated behaviours, weighs heavily on the industry [37]. The need for the development of enrichment opportunities has previously been expressed with some urgency, but research surrounding the efficacy and viability of options has been lacking [13]. This study highlights how a simple setup of enrichment options can change the overall usage and effect within a herd. The application of this could be to use low-hanging setups for mineral blocks in housed cattle to maximize uptake. However, the founding idea behind the study could be applied to enrichment items already in practice or items currently undergoing research. In either situation, it should be considered to what extent the enrichment has biological relevance and facilitates species-specific behaviour [6].

In future studies, variables such as health and production status, lameness, or the social dynamics of the herd should be evaluated as they could have influenced the extent of variability in the present study. A milk yield depression of 1.9 kg/head per day has been seen in cattle consuming large quantities of saline water [47], which may affect the practicality of salt block enrichment. However, the effect of ad lib salt and fresh water on milk yield has not been evaluated and prior studies have shown that higher milk yields require larger volumes of water intake [48], which could be supported by cows licking the salt and then drinking.

This study demonstrated that specific cattle will use the salt blocks more frequently and consistently. In the wild, animals visit natural salt deposits for nutritional and health benefits [49]. If a connection was found between overall health and the usage of the blocks as a luxury behaviour, which was seen in heifers offered brushes [50] and lame dairy cattle [51], it could lead to novel strategies for lameness and disease detection. Allowing for prompt veterinary intervention and possibly decreases in overall losses.

It should also be noted that the data collection methodology and study setup did not assess outcome-based husbandry. Enhanced forms of enrichment do not just address stereotypies or surface-level welfare concerns, but strive to create an interactive experience for the animals, which pushes them to use a multifaceted behavioural approach to the enrichment. The target and measure of success for outcome-based husbandry is the relevance of behaviour and appropriate responses to stimuli over time to support natural behaviours [12]. This approach could be implemented for future welfare studies and creating more targeted setups for the salt licks. In future research, it would be useful to explore breed variation and to look at dry as well as lactating cattle in terms of their preferences for the mounting of setup for salt blocks.

### 4.4. Limitations

This project was conducted on an active and expanding dairy farm, which led to limitations within the study. Active construction occurred during the last two weeks of observation, which may have influenced cattle behaviour due to increased environmental disturbance and potential stress. Also, as previously noted in the methods section, this construction forced a movement of the cattle during one of the study days and therefore data could not be collected that day. This was accounted for in the statistics; however, it did lead to a loss of data points. Data points were limited due to the time restraints of the study and the usage of a single farm. Future research would benefit from increased observation lengths for each mounting design and the inclusion of multiple UK dairy farms to further generalize the findings and ensure their applicability across different farming setups. This would also allow for further investigation into habituation patterns related to the enrichment items.

Along with this, only a single observer was used to collect and analyse the data, rather than 24-h CTV camera footage. Therefore, despite the large number of hours observed, the usage of the blocks outside of this time was not evaluated. In future, analysis of the day-long usage of blocks paired with environmental factors such as stress and heat should be considered.

Lastly, factors such as the stage of production, milk yield, parity, and health status of the cows were not considered. It would be beneficial for future research to investigate the influence these factors may have on engagement with enrichment and agonistic behaviours. It would also be pertinent to see if milk yield production is positively impacted by the usage of LM salt lick blocks compared to other mounting techniques.

## 5. Conclusions

This study demonstrated that the design of enrichment items does affect overall interaction and effectiveness within a dairy herd. Cattle increased new interactions with the salt licks when these were set up at a lower height compared to a hanging block at shoulder level. It was also seen that more cattle used the blocks and consumed more of the blocks when they were free hanging at a low height. Therefore, providing low-hanging (30 cm from the ground) movable salt licks shows promise as an effective form of environmental enrichment for lactating dairy cattle.

## Figures and Tables

**Table 1:** Description of the salt lick mounting setups for the study.

Treatment Name	Treatment Abbreviation	Height of Salt Lick from Ground	Movable	Week of Study
Low non-moveable	LNM	30 cm	No	1 and 4
Low moveable	LM	30 cm	Yes	2
High moveable	HM	150 cm	Yes	3

**Table 2:** Definitions of Behavioural Terminology.

Term	Definition
Interaction	The oral manipulation of a salt block by a cow
New Interaction	An interaction by a cow who was not observed at that block during the previous observation point
Individual Cow Interaction	The individual cow identified by ID tags who has partaken in an interaction
Total Individual Cow Interactions	The total number of individual cows, identified by ID tags, who have partaken in an interaction on a specific day

**Table 3:** Individual Cow Usage.

Cow ID	Number of Days a Block Was Used	Percentage of Days a Block Was Used (n = 20)
620	17	85%
413	16	80%
612	16	80%
665	16	80%
630	15	75%

**Table 4:** Individual Cow Interactions Over the Study.

Cow ID	Total Cow Interactions
620	118
413	117
612	89
665	88
630	82

**Table 5:** Instances of Aggression.

Week	Block Setup	Instances of Aggression	Description
1	LNM	13	Head-butting, Shoving, and Chasing
2	LM	4	Head-butting and Shoving
3	HM	1	Head-butting
4	LNM (repeat)	3	Head-butting and Chasing

## Data Availability

Data are available on request from the corresponding author.

## Abbreviations

The following abbreviations are used in this manuscript:

LNM	Low Non-Moveable Mounting Setup
LM	Low Moveable Mounting Setup
HM	High Moveable Mounting Setup
DEFRA	Department for Environment Food and Rural Affairs
